# Injury risk functions for the four primary knee ligaments

**DOI:** 10.3389/fbioe.2023.1228922

**Published:** 2023-10-04

**Authors:** Jiota Nusia, Jia-Cheng Xu, Johan Knälmann, Reimert Sjöblom, Svein Kleiven

**Affiliations:** ^1^ Department of Traffic Safety and Traffic Systems, The Swedish National Road and Transport Research Institute (VTI), Stockholm, Sweden; ^2^ Division of Neuronic Engineering, KTH Royal Institute of Technology, Stockholm, Sweden; ^3^ Department of Strength and Crash Analysis, Scania CV AB, Södertälje, Sweden

**Keywords:** injury risk function, knee ligaments, cruciate ligament, collateral ligament, failure strain, human body model, cumulative distribution function

## Abstract

The purpose of this study was to develop injury risk functions (IRFs) for the anterior and posterior cruciate ligaments (ACL and PCL, respectively) and the medial and lateral collateral ligaments (MCL and LCL, respectively) in the knee joint. The IRFs were based on post-mortem human subjects (PMHSs). Available specimen-specific failure strains were supplemented with statistically generated failure strains (virtual values) to accommodate for unprovided detailed experimental data in the literature. The virtual values were derived from the reported mean and standard deviation in the experimental studies. All virtual and specimen-specific values were thereafter categorized into groups of static and dynamic rates, respectively, and tested for the best fitting theoretical distribution to derive a ligament-specific IRF. A total of 10 IRFs were derived (three for ACL, two for PCL, two for MCL, and three for LCL). ACL, MCL, and LCL received IRFs in both dynamic and static tensile rates, while a sufficient dataset was achieved only for dynamic rates of the PCL. The log-logistic and Weibull distributions had the best fit (*p*-values: >0.9, RMSE: 2.3%–4.7%) to the empirical datasets for all the ligaments. These IRFs are, to the best of the authors’ knowledge, the first attempt to generate injury prediction tools based on PMHS data for the four knee ligaments. The study has summarized all the relevant literature on PHMS experimental tensile tests on the knee ligaments and utilized the available empirical data to create the IRFs. Future improvements require upcoming experiments to provide comparable testing and strain measurements. Furthermore, emphasis on a clear definition of failure and transparent reporting of each specimen-specific result is necessary.

## 1 Introduction

Knee ligament injuries are associated with both low-energy and high-energy trauma (Schlumberger et al., 2020). Traffic- and sport-related accidents (high- and low-energy trauma, respectively) are the two main causes of anterior and posterior cruciate ligament injuries (ACL and PCL, respectively), leading to primary reconstructions, both as single injuries and multi-ligament injuries (Nicolini et al., 2014; Owesen et al., 2018; Prentice et al., 2018; The Swedish National Knee Ligament Registry, 2019). A recent study on US injury data by Mallory et al. (2022) found pedestrians to be subjected to knee ligament injuries during accidents while using motor vehicles. Among the adult pedestrians (16+ y/o) sustaining knee ligament injuries with no adjacent fractures, approximately 38% were distributed to pure collateral ligament injuries, 31% to cruciate ligament injuries, and 31% sustained injuries on both ligament groups. Similar distributions were also found for ligament injuries involving knee-adjacent fractures. These results indicate that knee ligament injuries are present problems in pedestrian–vehicle collisions and that the relative loading of the knee ligaments could depend on impact conditions such as the relative knee–vehicle bumper height and knee orientation at the time of impact (Mallory et al., 2022).

Although not life-threatening, knee ligament injuries increase the risk of subsequent injuries such as arthritis, meniscus tear, and the need for a total knee replacement (Sanders et al., 2017a; Sanders et al., 2017b; Sepúlveda et al., 2017; Wang et al., 2018). They also reduce the possibility of returning to previous levels of sporting activity (Sepúlveda et al., 2017; Everhart et al., 2018; Nwachukwu et al., 2019) and can have a long-term negative effect on the quality of life (Filbay et al., 2015; Filbay et al., 2018).

The four primary knee ligaments, ACL, PCL, and the medial and lateral collateral ligaments (MCL and LCL, respectively), are commonly associated with three different injury mechanisms: 1) mid-section failure is a rupture within the ligament itself, 2) ligament detachment occurs at the interface between the ligament and the bone, and 3) avulsion fractures occur when osseous fragments adjacent to the ligament insertion sites detach together with the ligament (Noyes and Grood, 1976; Lee and Hyman, 2002; Robinson et al., 2005; Paschos et al., 2010; White et al., 2013; Winkelstein, 2013; Marieswaran et al., 2018; Cho and Kwak, 2020). These three failure modes are a consequence of the interaction between the ligament and bone due to their substantially dissimilar mechanical properties.

The risk of injury can be evaluated using numerical simulations. Finite-element human body models (HBMs), such as Total HUman Model for Safety (THUMS) and the Global Human Body Model Consortium (GHBMC), are expected to increasingly complement experiments with physical dummies. HBMs offer the opportunity to evaluate impact down to the tissue level and address occupant diversities to a greater extent than what is practically possible with physical dummies. Local injury risk functions (IRFs) are needed in the evaluation of knee ligament responses as they predict the risk of injury on a material level, allowing injury mechanisms to be accounted for. Existing human-based IRFs for the lower extremities have primarily been focusing on skeletal fractures (Kuppa et al., 2001; Laituri et al., 2006; Prasad et al., 2010; Rupp et al., 2010; Weaver et al., 2015; Yoganandan et al., 2015), while IRFs for knee ligaments based on human data are missing from the literature. These lower extremity IRFs describe the global injury risk of the knee–thigh–hip (KTH) complex (Kuppa et al., 2001; Laituri et al., 2006; Prasad et al., 2010; Rupp et al., 2010) and assume injury risk based on only fracture loads. They, therefore, do not address the knee ligament responses. Knee ligament injuries are dependent on impact locations, and the IRFs herein might underestimate the risk for KTH injury by not being sensitive enough to capture knee ligament injuries caused by impact loads below fracture magnitudes (Prasad et al., 2010).

Presently, there are no IRFs for any of the four knee ligaments. As every ligament has a unique role in stabilizing the knee joint, it is essential to address the ligaments separately. Therefore, local IRFs of the knee ligaments are needed tools in order to understand the impact responses of each ligament, which would further enhance the potential of using HBM simulations to develop preventive measures. The objective of this study is to map the available literature for PHMS experimental tensile failure studies of the four primary knee ligaments: ACL, PCL, MCL, and LCL, and use the obtained literature data to create local IRFs.

## 2 Methods

Cumulative injury risk functions were derived from specimen-specific failure strains in experimental studies conducted on post-mortem human subject (PMHS) ligaments as previously utilized for hip fracture risk functions by Kleiven. (2020). However, most results were provided as averaged failure strains, and only a limited number of individual specimen-specific strain results were found in the literature, entailing insufficient sample information to alone construct IRFs. Therefore, the focus was shifted to utilizing available literature data by statistically generating failure strains from the provided mean and standard deviation (SD).

### 2.1 Study search

A literature search was performed for experimental studies conducting uniaxial failure tests on PMHS ligaments. The search was conducted iteratively between February 2019 and April 2023, mainly using Google Scholar. Some of the search words used included “ACL/PCL/MCL/LCL material properties,” “Failure strain,” “Tensile properties,” and “Knee joint” in various combinations. Most of the collected studies shown in Supplementary Table A1 in Supplementary Appendix A were found by reviewing the reference lists in articles generated by the Google Scholar search.

### 2.2 Study selection

The inclusion criteria of studies for the injury risk functions were as follows: 1) ligament failures (deformation at maximum load) presented in terms of strain values or elongation failures together with initial ligament lengths; 2) conducted on adult PMHS; and 3) primary sources of the experimental results, exclusively. The results stating bony fracture or avulsion as the failure mode were excluded as they do not represent an injury mechanism on the ligament tissue itself. Table 1 lists all studies meeting these criteria.

**TABLE 1:** List of studies used to construct the injury risk functions, presenting each corresponding mean failure strain ±SD. The uniaxial tensile tests were conducted on either bone–ligament–bone (BLB) specimens or dissected LIGaments (LIG). The studies are grouped according to dynamic (red) or static (blue) tensile rate. “N” represents the number of specimens used in the averaging, and “Failure mode” specifies the injury mechanisms of the BLB specimens; 1) mid-substance ligament failure or 2) failure at ligament attachment site. Empty boxes indicate non-provided information. *Specimen-specific results provided.

Study	Mean strain [%]	SD [%]	N	Tensile rate	Specimen	Failure mode
Anterior cruciate ligament						
Butler et al. (1992)—AMB*	19.1	2.8	5	100%/s	BLB	Ligament and insertion site
Butler et al. (1992)—ALB*	16.1	3.9	6	100%/s	BLB	Ligament and insertion site
Butler et al. (1992)—PC*	15.2	5.2	6	100%/s	BLB	Ligament and insertion site
Chandrashekar et al. (2006)—males	30.0	6.0	8	100%/s	BLB	Ligament failure
Chandrashekar et al. (2006)—females	27.0	8.0	9	100%/s	BLB	Ligament failure
Nooyes and Grood (1976)—younger	44.3	8.5	6	100%/s	BLB	Ligament failure
Marieswaran et al. (2021)*	45.0	3.0	6	300%/s	BLB	Insertion site
Marieswaran et al. (2021)*	42.0	2.0	6	30%/s	BLB	Ligament and insertion site
Van Dommelen et al. (2005)—aACL	18.0	2.8	4	54% ± 9.2%/s	BLB	Ligament failure
Van Dommelen et al. (2005)—pACL	22.0	3.0	3	63% ± 3.4%/s	BLB	Ligament failure
Kennedy et al. (1976)	30.8	2.3	10	2.083 mm/s	LIG	
Kennedy et al. (1976)	35.8	2.8	10	8.33 mm/s	LIG	
Paschos et al. (2010)*	42.7	18.5	10	1.5 mm/s	BLB	Ligament and insertion site
Marieswaran et al. (2021)*	44.0	3.0	6	3%/s	BLB	Insertion site
Posterior cruciate ligament						
Butler et al. (1986)—donor 1	14.6	4.6	3	100%/s	BLB	Ligament failure
Butler et al. (1986)—donor 2	14.0	2.4	2	100%/s	BLB	Ligament failure
Butler et al. (1986)—donor 3	18.9	2.9	3	100%/s	BLB	Ligament failure
Race and Amis (1994)—aPCL	18.0	5.3	7	50%/s	BLB	
Race and Amis (1994)—pPCL	19.5	5.4	10	50%/s	BLB	
Van Dommelen et al. (2005)—aPCL	18.0	2.3	2	45% ± 5.7%/s	BLB	Ligament failure
Van Dommelen et al. (2005)—pPCL	14.0	1.4	3	49% ± 5.3%/s	BLB	Ligament failure
Kennedy et al. (1976)	28.3	1.9	10	2.083 mm/s	LIG	
Kennedy et al. (1976)	24.2	2.1	10	8.33 mm/s	LIG	
Medial collateral ligament						
Kerrigan et al. (2003)*	11.5	5.3	3	1,205% ± 306%/s	BLB	Ligament failure
Kennedy et al. (1976)	24.3	1.3	10	8.33 mm/s	LIG	
Kennedy et al. (1976)	23.0	2.4	10	2.083 mm/s	LIG	
Kerrigan et al. (2003)*	20.3	3.9	3	1.78% ± 0.35%/s	BLB	Ligament failure
Quapp and Weiss (1998)	17.1	1.5	9	1%/s	LIG	
Smeets et al. (2017)*	22.9	2.5	12	2%/s	LIG	
Lateral collateral ligament						
Butler et al. (1986)—donor 1	10.5	2.5	2	100%/s	BLB	Ligament failure
Butler et al. (1986)—donor 2	12.7	0.9	2	100%/s	BLB	Ligament failure
Butler et al. (1986)—donor 3	16.7	3.2	2	100%/s	BLB	Ligament failure
Kerrigan et al. (2003)*	10.5	4.6	3	1908%/s	BLB	Ligament failure
LaPrade et al. (2005)	16.0	5.0	8	100%/s	BLB	Ligament failure
Wilson et al. (2012)*	12.5	2.8	9	20%/s	BLB	Ligament and insertion site
Smeets et al. (2017)*	41.0	9.9	11	2%/s	LIG	
Sugita and Amis (2001)	16.1	2.5	9	3.33 mm/s	BLB	
Van Dommelen et al. (2005)	15.0	2.9	4	≤0.001%/s	BLB	Ligament failure
Van Dommelen et al. (2005)	20.0	5.5	6	0.04% ± 0.009%/s	BLB	Ligament failure

Five articles provided specimen-specific failure strains (Butler et al., 1992; Kerrigan et al., 2003; Paschos et al., 2010; Smeets et al., 2017; Marieswaran et al., 2021). Wilson et al. (2012) presented the failure elongations for each LCL specimen; however, the initial lengths were given as an average. Virtual failure strains were, therefore, statistically generated based on the given mean failure strain. Van Dommelen et al. (2005) included the results of Kerrigan et al. (2003) in the averaging of the LCL failure strains. Hence, to avoid duplication of data, the reported LCL results in Kerrigan et al. (2003) were not applied. Furthermore, the results given by van Dommelen et al. (2005) for MCL were excluded from the current study due to the declared use of an inaccurate initial MCL length in the failure strain calculation. Paschos et al. (2010) strain results at the maximum tensile load were extracted from the force-elongation graphs using the online tool WebPlotDigitizer (Rohatgi, 2020).

### 2.3 Categorization of the dataset

The injury risk functions were generated based on whether the experiments were conducted on bone–ligament–bone (BLB) specimens or on dissected LIGament samples (LIG), as the two specimen types differ in which of the failure mechanisms they employ.

**FIGURE 1:**
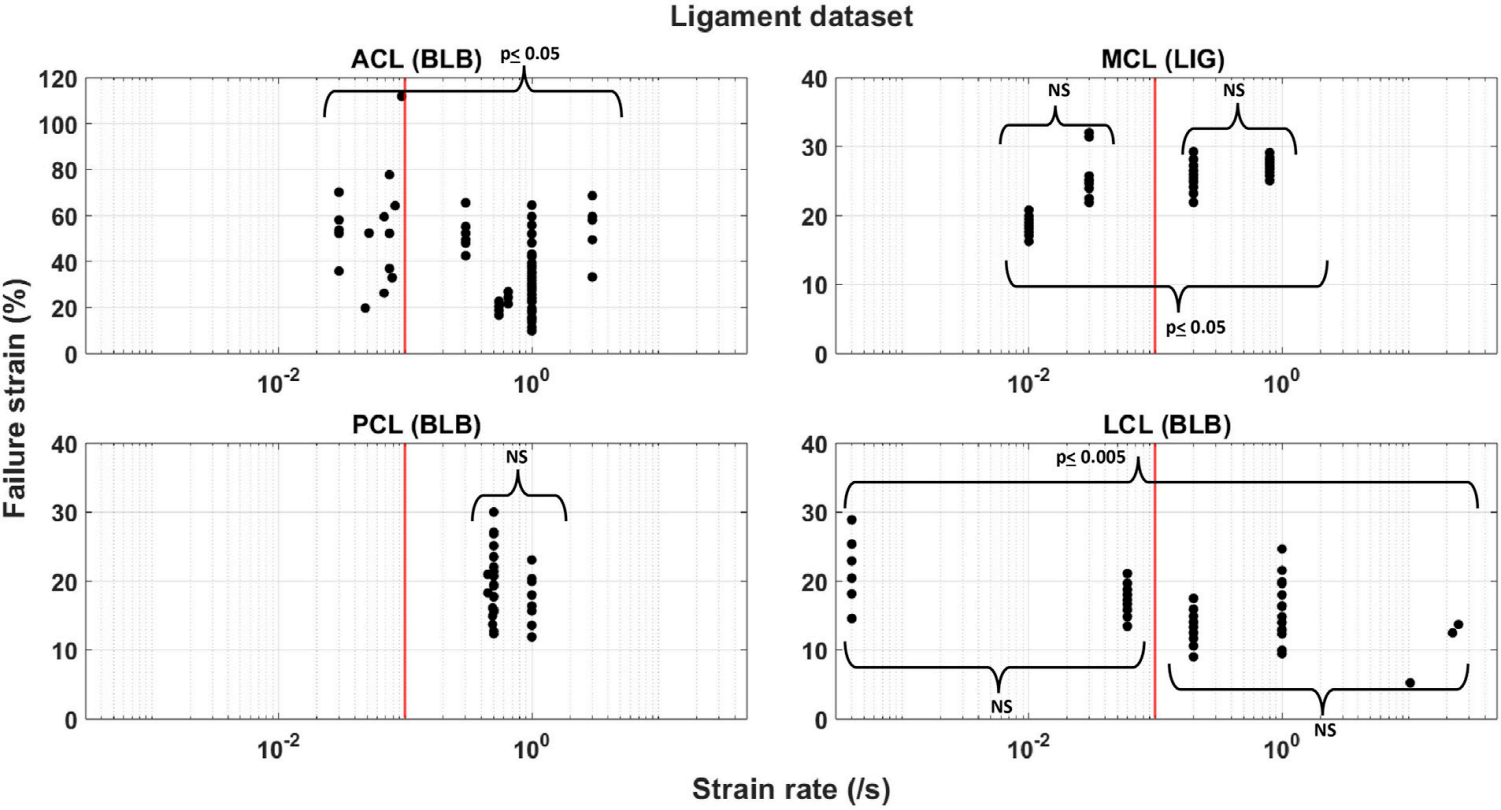
Distribution of the ligament dataset on a logarithmic strain rate scale between the two categorized groups of “static” and “dynamic” at a cutoff strain rate of 10%/s (red line). Student’s t-test was conducted within and between each group at a significance level of 5%. NS denotes no significance.

To find an appropriate categorization in the wide range of tensile rates, the failure strains were grouped together in a (1, 10, 100, and 1,000) %/s order of magnitude. The four groups were systematically tested among themselves for statistical significance in the strain rate using Student’s t-test. Significant differences (*p* ≤ 0.05) in the failure strains were found at a mutual cutoff level of 10%/s for all ligaments (Figure 1). Statistical significances were, thereafter, tested for the failure strains below the cutoff level against the failure strains above the cutoff level. Injury risk functions were, therefore, constructed based on two tensile rate groups. Rates below 10%/s were grouped together and labeled as “static,” and rates equal to, or above, the cutoff level were labeled as “dynamic.”

**FIGURE 2:**
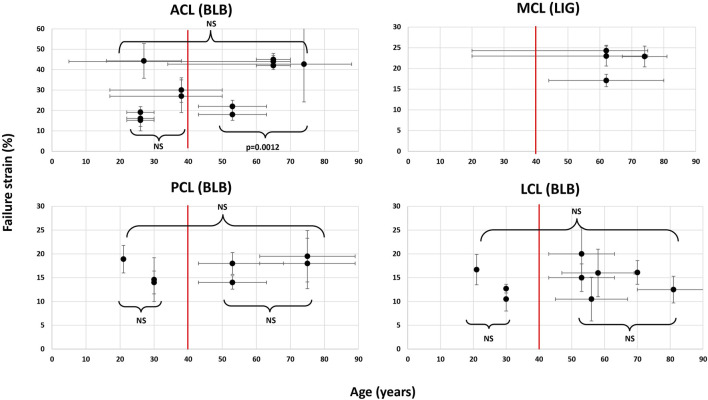
Distribution of the mean failure strains relative to the mean donor ages from each data source found in Table A1, Appendix A. The vertical and horizontal error bars represent the range or ±SD to the corresponding mean age and mean failure strain, respectively. The figure illustrates an example of Student’s t-test (*p* ≤ 0.005) conducted within and between two groups at a cutoff age (red line) of 40 years. NS denotes no significance.

The failure strains seen in Table 1 are results based on specimens with varied donor ages. Most of the studies have presented the averaged, and not the specimen-specific, age of their specimens (Supplementary Table A1 in Supplementary Appendix A). Student’s t-test was conducted to estimate the significance of the mean failure strains between subgroups of the specimens (Figure 2). No significance was found for any of the ligaments, and the dataset was, therefore, not further divided based on the donor age.

**FIGURE 3:**
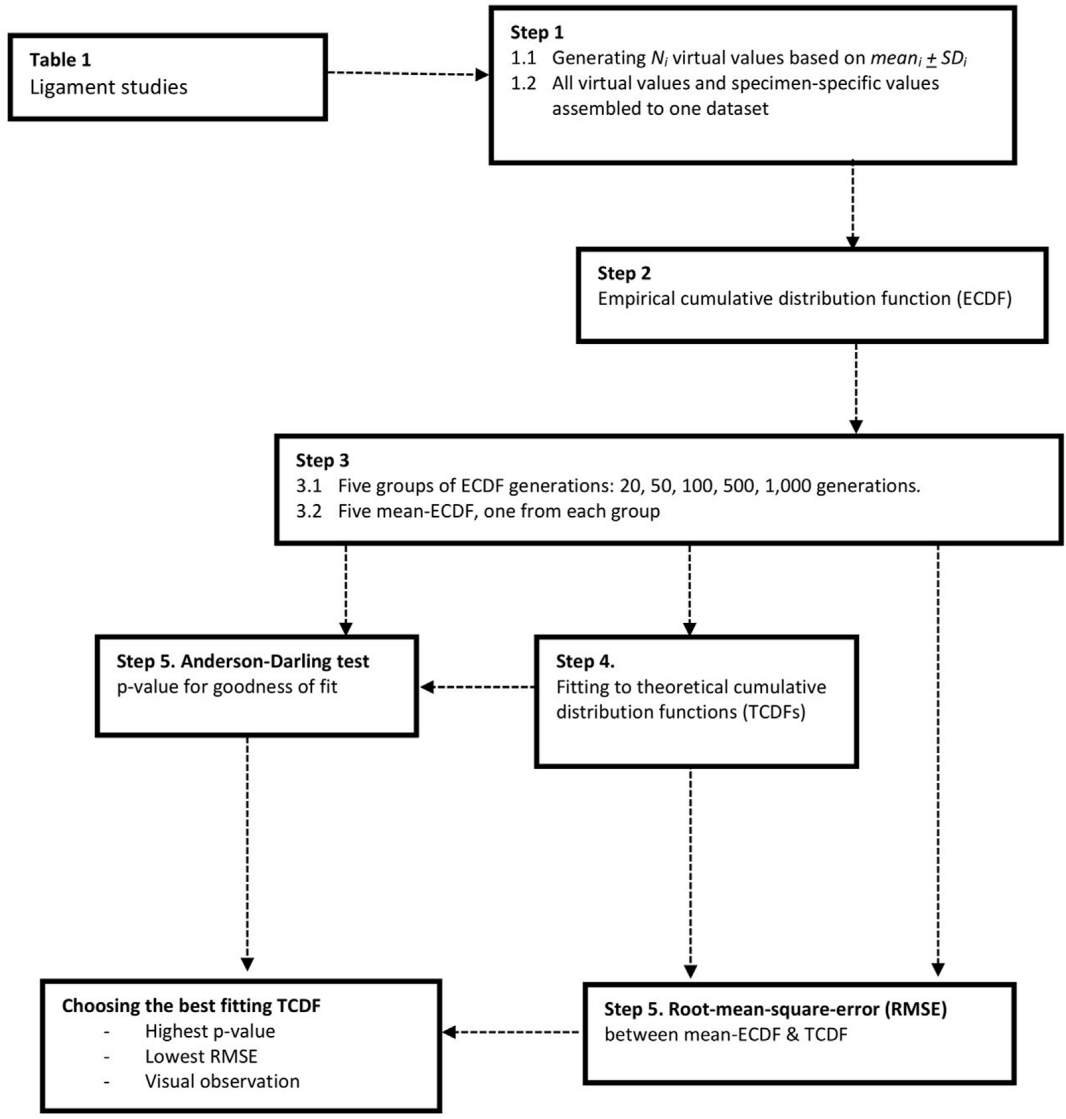
The process of constructing the injury risk functions. Studies that did not provide specimen-specific failure strains had experimental values statistically generated based on mean ± SD (Step 1). The virtual values of all studies were thereafter assembled together with the provided specimen-specific results and collectively represented the dataset for one risk function (Step 2). Five groups, composed of 20–1,000 generations of ECDFs, were generated. Each group received an averaged ECDF, giving a total of five mean ECDFs (Step 3). Each mean ECDF was tested against various theoretical distributions, generating one each corresponding TCDF (Step 4). The best fitting TCDF was chosen based on the theoretical distribution’s goodness of fit using the Anderson–Darling test, on the root mean square error (RMSE) between the mean ECDF and on the TCDF and visual observations (Step 5).

IRFs were generated for sample sizes of at least 10 failure strains. Table 2 gives an overview of their characteristics, and Figure 3 illustrates the construction procedure of the IRFs.

**TABLE 2:** Ten injury risk functions (IRFs) were generated representing either bone–ligament–bone (BLB) specimens or dissected LIGament samples (LIG), studied at a tensile rate either below (“static”) or equal and above (“dynamic”) 10%/s. The IRFs were composed of either statistically generated values (denoted “virtual”) or specimen-specific values, or a mix of the two.

Ligament	Rate	Specimen	Failure strain values	Sample size
ACL	Dynamic	BLB	Mix	59
ACL	Static	BLB	Specimen-specific	16
ACL	Dynamic	LIG	Virtual	20
PCL	Dynamic	BLB	Virtual	30
PCL	Dynamic	LIG	Virtual	20
MCL	Dynamic	LIG	Virtual	20
MCL	Static	LIG	Mix	21
LCL	Dynamic	BLB	Mix	26
LCL	Static	BLB	Virtual	19
LCL	Static	LIG	Specimen-specific	11

### 2.4 Generating virtual values

The failure strains used for the generation of virtual values (Table 1) were assumed to be normally distributed as the results were presented as mean and SD only. If not stated otherwise, all strains were, furthermore, assumed to be engineering strains and were converted to Green–Lagrange strains ( ε ). Eq. 1 and the Box–Müller basic transform (Box and Muller, 1958) were adopted for the generation of normally distributed virtual values, Eq. (2):

$$\varepsilon =\frac{1}{2}\left(\lambda^{2}-1\right),$$ (1)

$$Z=\sqrt{-2\ln\left(U_{1}\right)}*\;\cos\left(2\pi U_{2}\right),$$ (2)

$$X_{virtual}=Z*\varepsilon_{SDi}+\varepsilon_{MEANi},$$ (3)

where λ is the stretch ratio. *U*_*1*_ and *U*_*2*_ are two series of independent random variables, uniformly distributed in the interval [0,1] and corresponding to each study’s sample size *N* (Table 1). *Z* is a set with the resulting independent random variables having a normal distribution, which was then shifted to match each study’s mean failure strain and SD, Eq. (3). Conclusively, X_virtual_ is a collection of *N* statistically estimated failure strains, corresponding to the study’s $\varepsilon_{MEANi}$ and $\varepsilon_{SDi}$ . All X_virtual_ from each study *i* (including the specimen-specific results, if provided) were assembled in one dataset to derive one injury risk function.

### 2.5 Constructing injury risk functions

As the values in X_virtual_ were randomly generated failure strains within the range of every $\varepsilon_{MEANi}$ and $\varepsilon_{SDi}$ , the robustness of the method was evaluated by generating groups of 20, 50, 100, 500, and 1,000 empirical cumulative distribution functions (ECDFs) for each ligament. The analysis was based on the calculated mean ECDFs representing each group of ECDF generation with respect to the injury risk. Theoretical (parametric) distributions commonly used within survival analysis (George et al., 2014) were fitted against the mean-ECDF from all five groups by using the MATLAB function “fitdist,” and the goodness of fit was evaluated with the Anderson–Darling test (AD test; Anderson and Darling, 1952). The best fitting probability distribution function was chosen to derive the cumulative distribution function, to define the risk of failure based on strain. One of the theoretical cumulative distribution functions (TCDFs) was chosen to formulate each injury risk function. The evaluation was based on 1) the largest *p*-values from the AD test, 2) the lowest root mean square error (RMSE) between the mean-ECDF and each distribution’s corresponding TCDF, and 3) visual observation of the plotted curves (Figure 3). The visual observations aimed to control for good fit primarily for the lower levels of injury risk, as they are of most relevance in injury evaluation. The parameters in the cumulative distribution function of the chosen theoretical distribution were defined based on the smallest confidence interval between the five generation groups.

## 3 Results

Fifteen publications met the inclusion criteria for the generation of 10 IRFs. Of the tested distributions, the log-logistic and Weibull distributions showed the best fit to the empirical datasets. Table 3 summarizes the resulting *p*-values and RMSEs for the chosen generation groups of the best fitting distributions, having most *p*-values ranging above 0.9 and RMSEs between 2.3% and 4.7%, respectively.

**TABLE 3:** Log-logistic and Weibull shape and scale parameters for the 10 IRFs representing the cruciate (ACL and PCL) and collateral (MCL and LCL) ligaments. The 95% confidence intervals are presented within brackets. The resulting *p*-value for the goodness of fit in the Anderson–Darling test and the root mean square error (RMSE) is given for the best fitting distribution of the chosen generation groups.

Injury risk function	Distribution	Scale parameter, *α*	Shape parameter, β	*p*-value	RMSE (%)	Generation group
ACL-dynamic-BLB	Log-logistic	29.87 (26.09–34.20)	3.46 (2.79–4.30)	0.851	4.04	100
ACL-static-BLB	Log-logistic	50.28 (40.64–62.2)	4.14 (2.71–6.32)	0.907	7.74	-
ACL-dynamic-LIG	Log-logistic	38.67 (36.79–40.65)	15.74 (11.05–22.43)	0.976	4.70	100
PCL-dynamic-BLB	Log-logistic	18.62 (16.88–20.54)	6.39 (4.76–8.59)	0.999	2.33	500
PCL-dynamic-LIG	Weibull	31.15 (29.83–32.53)	10.68 (7.58–15.05)	0.995	4.68	500
MCL-dynamic-LIG	Log-logistic	21.52 (19.61–23.62)	8.80 (6.04–12.82)	0.908	4.83	50
MCL-static-LIG	Weibull	27.40 (26.57–28.22)	15.37 (10.94–21.61)	1	3.07	100
LCL-dynamic-BLB	Log-logistic	13.90 (12.40–15.59)	5.65 (4.11–7.78)	0.999	2.59	500
LCL-static-BLB	Log-logistic	18.12 (16.41–20.01)	7.90 (5.43–11.48)	1	2.86	100
LCL-static-LIG	Log-logistic	40.21 (35.47–45.58)	8.58 (5.11–14.42)	0.971	10.2	-

**FIGURE 4:**
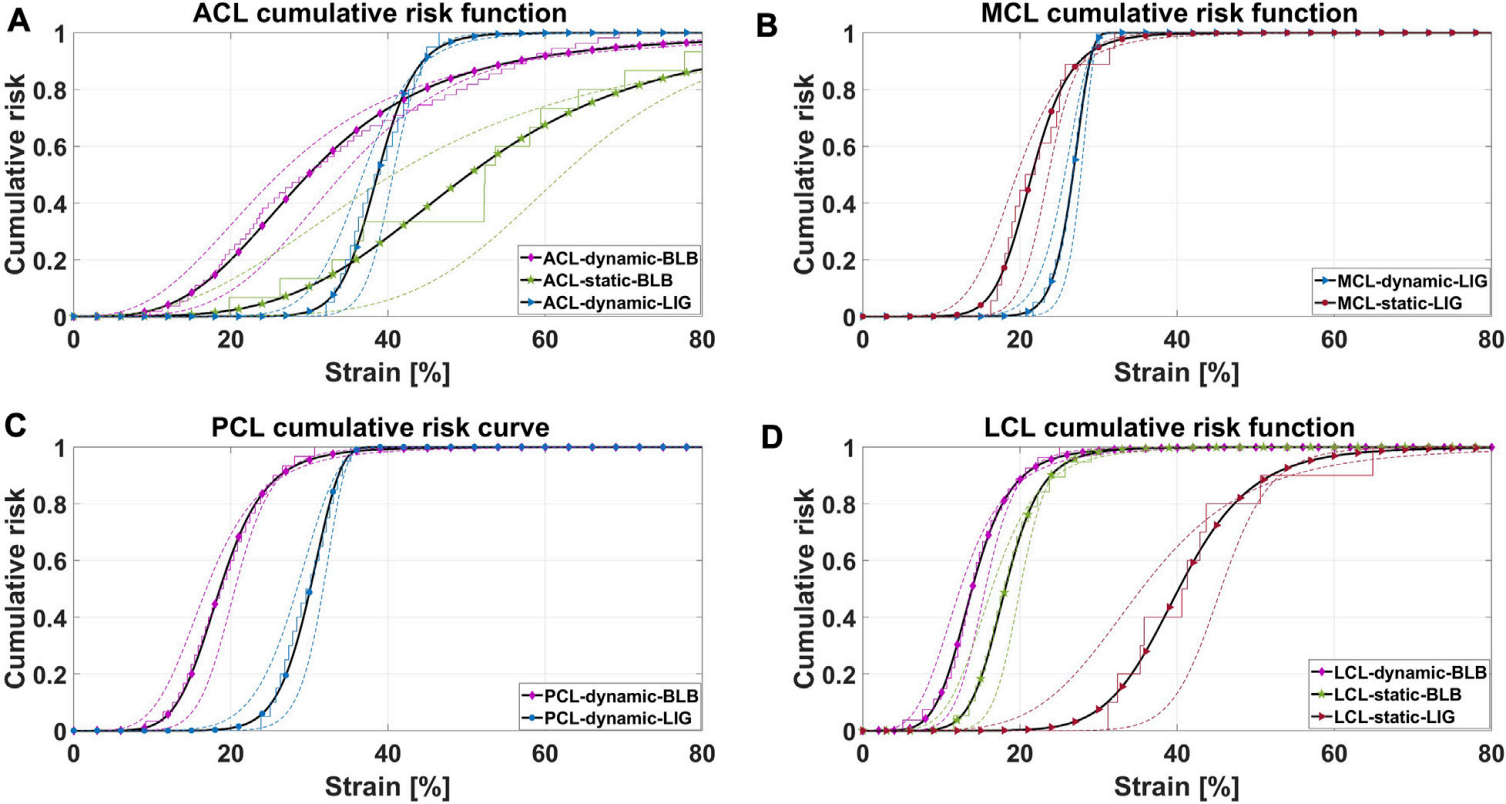
Ten cumulative injury risk functions (IRFs) with 95% confidence intervals (dotted lines), generated from the corresponding mean ECDF. Derived from failure strains of PMHS ACL, PCL, MCL, and LCL specimens, the IRFs are representing either bone–ligament–bone (BLB) or dissected LIGament preparations (LIG), tested in either dynamic or static rate. One experimental data point in the ACL-static-BLB mean ECDF reached 112% failure strain and is, therefore, not visualized in **(A)**. **(A)**. Anterior cruciate ligament injury risk functions. **(B)** Medial collateral ligament injury risk functions. **(C)** Posterior cruciate ligament injury risk functions. **(D)** Lateral collateral ligament injury risk functions.

All ACL and PCL IRFs had slightly higher *p*-values and/or lower RMSEs for the gamma or log-normal distributions than the chosen distributions (Supplementary Table B1 in Supplementary Appendix B). However, the visual observations found the differences in the fit to be mainly located in the upper end of all the IRFs (above 60% of risk), whereas the lower end fitted equally well or better for the log-logistic or Weibull distributions. As the lower end of an IRF is more applicable for injury prevention, the two distributions were selected because of the simplicity of their IRFs. The IRFs of Weibull and log-logistic distributions are expressed in Eq. (4) and (5), respectively, and shown in Figure 4. Table 3 presents the resulting parameters for the chosen groups of the ECDF generations.

$$\text{Weibull CDF}:F\left(\varepsilon \right)=1-e^{{-\left(\varepsilon /\alpha \right)}^{\beta }},$$ (4)

Log−Logistic CDF:Fε=εβεβ+αβ, (5)

where ε is the Green–Lagrange strain, *α* is the scale parameter, and *β* is the shape parameter.

Student’s t-test analysis confirmed a statistical difference between the datasets of the static and dynamic subgroups of ACL BLB and MCL LIG (*p* < 0.001, respectively) and for LCL BLB (*p* = 0.002) at a significance level of 5%.

## 4 Discussion

To address current gaps in the literature, this study provides risk functions for the four primary knee ligaments. Ten injury risk functions were derived based on mean failure tensile strains conducted on PMHS ACL, PCL, MCL, and LCL specimens. ACL and LCL have one IRF representing each of the BLB and LIG specimen types, both for the dynamic and static subgroups. To date, an insufficient number of studies have met the inclusion criteria to generate static IRFs for PCL and bone–ligament–bone IRFs for MCL.

### 4.1 The injury risk functions

The wide corridors of ACL-static-BLB and LCL-static-LIG are partly a consequence of the small dataset composing these ECDFs (16 and 11 data points, respectively) and the large SD, which is also reflected on the RMSEs of about 8% and 10%, respectively. The ACL-dynamic-BLB IRF has a slower increase of risk than the other dynamic IRFs (Figure 4), as a result of the wide range of failure strains (15%–45%) composing the ECDF. Furthermore, the mean ECDF of ACL-dynamic-BLB (Figure 4A) does not align perfectly with the TCDF. These failure strains are conducted with strain rates ranging from 30%–300%/s, on specimens with donor age across the whole lifespan (Table 1 and Supplementary Table A1 in Supplementary Appendix A), which could have influenced the diverse outcomes. The dynamic failure strains by Marieswaran et al. (2021) are notably higher than the rest of the dataset. The authors compared their results to those of Chandrashekar et al. (2006), whose failure strains were about 30%, and analyzed that the differences in the experimental setup could have been one cause of the diverse outcomes. While Marieswaran et al. (2021) pulled the ACL at a zero degree of knee flexion, Chandrashekar et al. (2006) positioned it at 45 degrees of flexion. The larger knee angle was analyzed to have caused a pre-stretch of the whole ACL, which generated comparably lower failure strains (also observed in Table 1).

There are a number of limitations in comparing the ligament tensile responses between different knee angles (also discussed in Section 4.3). The orientation of the two bundles of the ACL varies inside the knee joint, and loading the ligament in the longitudinal direction along both bundles can be considered challenging (Woo et al., 1991). Most tensile failure studies of the cruciate ligaments used BLB specimen preparations (Supplementary Table A1 in Supplementary Appendix A). Opposed to the PCL studies, multiple ACL studies (Noyes and Grood, 1976; Trent et al., 1976; Woo et al., 1991; Jones et al., 1995; Chandrashekar et al., 2006; Paschos et al., 2010; Marieswaran et al., 2018; Marieswaran et al., 2021) conducted the testing on the whole ligament, denoted as the femur–ACL–tibia complex (FATC), that is, not separating the ligament into anterior and posterior bundles. The FATC results used in the current study applied the load along the ACL axis (Noyes and Grood, 1976; Chandrashekar et al., 2006; Paschos et al., 2010; Marieswaran et al., 2021). Paschos et al. (2010) defined three different failure patterns of the ACL, based on the failure sequences of the bundles. Observing double peaks in the force-elongation graphs, Paschos et al. (2010) demonstrated the role of the ACL as a multifiber ligament, as the two bundles did not rupture simultaneously during loading. Woo et al. (1991) further observed that the structural properties and the failure modes (bone avulsion, ligament attachment site, and mid-substance failure) in the FATC were affected depending on the tensile load alignment along the ligament. The two knee orientations tested by Woo et al. (1991) showed both a difference in load uptake by the whole ligament and the uneven load distribution within the ACL, indicating that ACL failure is sensitive to knee orientation during uniaxial tensile tests. Considering that all the FATC experiments used in the current study present the largest failure strains compared to the other ACL studies (Table 1), there is reason to suspect an interaction between the two bundles, together increasing the structural integrity by picking up the load during failure. To control for this issue, concerning both cruciate ligaments, future experiments are suggested to measure the distribution of the tensile load between the posterior and anterior bundles.

The BLB IRFs of the dynamic ACL and PCL, and the static LCL, are positioned to the left, relative to their corresponding LIG IRFs, as a result of the BLB dataset having lower failure strains than the LIG dataset (Table 1). Both the PCL and LCL-BLB IRFs show a distinct separation compared to the IRFs of the dissected ligaments, having strains at mean risk of failure approximately double in magnitude (PCL-dynamic-BLB vs. PCL-dynamic-LIG and LCL-static-BLB vs. LCL-static-LIG). These results are in line with those of previous experiments conducted by Robinson et al. (2005), where similar relations were observed when comparing MCL failure loads between BLB complexes and dissected ligaments. Although no conclusions can be drawn from the aforementioned observations, it is possible that failure at attachment sites occurred prior to mid-substance failures.

The IRFs of the MCL are only based on dissected ligaments which are provided for both the dynamic group, as well as the static. While the BLB IRFs for the other three ligaments behave as expected, the MCL IRFs have a reversed and unexpected relation between the dynamic IRF and the static IRF. The dynamic IRFs for ACL, PCL, and LCL have lower strain failures and more rapid accelerations of the injury risks compared to the static rates (Table 1), positioning the dynamic IRFs to the left of the static IRFs. For the MCL, however, the dynamic failure strains are larger than the static failure strains (23% and 24.3% vs. 17.1% and 22.9%), which consequently arranges the dynamic IRF to the right of the static IRF. A reasonable explanation for this was not found when comparing the experimental setups between the three studies composing the MCL IRFs (Kennedy et al., 1976; Quapp and Weiss, 1998; Smeets et al., 2017). All three studies subjected the specimens to axial loading by clamping the ends of the specimens. While Quapp and Weiss (1998) and Smeets et al. (2017) tested on dog-bone-shaped specimens, Kennedy et al. (1976) appear to have used rectangular ones (with similar dimensions as the gauge dimensions in Quapp and Weiss (1998)). All studies pretensioned the ligaments before failure testing, but only Quapp and Weiss. (1998) and Smeets et al. (2017) preconditioned them as well. All studies calculated the engineering strain of the ligaments. Quapp and Weiss (1998) measured the failure strain using video analysis on black markers attached to the specimens, while Kennedy et al. (1976) used an optical extensometer and an oscillograph. Smeets et al. (2017) did not clearly state how the strain rates and failure strains were measured. However, failure was defined at the ultimate load, which indicates that the tensile apparatus provided the displacement metrics. Both the dynamic and static IRFs are approximately composed of the same number of specimens. The static IRF is mostly based on male donors (19 male and three female donors), while this information was not provided in the data used to construct the dynamic IRF. The dynamic IRF is based on a mix of younger and older specimens (ranging between 20 and 75 y/o), while the static IRF leans toward an older age span (62 ± 18 and 74 ± 7 y/o). Failure strains are believed to be influenced by age, where younger specimens are expected to be stronger than older specimens. However, given the combination of both the differences and the similarities in all the aforementioned variables, the rationale for the reversed relation between the dynamic and static IRFs of MCL was not identified.

### 4.2 Failure mechanisms and tensile rates

In the literature, data used in studies have tended to assume homogeneity of the ligaments. This assumption was therefore adopted in the construction of the IRFs. The defined tensile rate in most articles refers to the applied actuator displacement rate and not to the resulting ligament strain rate. That is, the experiments assumed that the displacement rate of the attachment grips corresponds to the ligament’s actual strain rate. This assumption might be applicable for the dissected ligaments; however, it is not obvious for the BLB components. As these specimens are a complex mix of bone and ligament, an additional variety of failure modes are possible when the ligament attachments are included in the test setup. The flaw of such simplification can be exemplified with ligament failures occurring at one attachment site and rarely in both, suggesting an inhomogeneous strain field across a ligament during uniaxial loading (Robinson et al., 2005; Paschos et al., 2010; Wijdicks et al., 2010; Cho and Kwak, 2020). Nevertheless, studies experimenting on dissected ligaments exclude the insertion site failure mode by taking the ligament out of its environmental context. However, they gain in precision by distinctively addressing only the mid-ligament failures. BLB complexes address both failure modes, although the challenge of linking the failure strain to a specific injury mechanism increases. Some studies suggest that failure at the attachment sites occurs prior to mid-ligament failure (Noyes and Grood, 1976; Robinson et al., 2005). Other studies indicate the rate-dependence of the failure modes (Lee and Hyman, 2002; Van Dommelen et al., 2005), which supports the requirement that in future studies local failure strains should address different knee ligament injury mechanisms.

### 4.3 The knee joint kinematics

The tensile recruitment of the knee ligaments is highly dependent on the position of the knee joint at the time of impact or injury. The cruciate ligaments are divided into two functional bundles with a varied orientation relative to each other within the knee joint. The ACL is divided into an anteromedial (AM) and posterolateral (PL) bundle, and PCL into an anterolateral (AL) and posteromedial (PM) bundle. The varied orientation of the bundles makes them load-bearing in different knee joint angles. The AM-ACL and AL-PCL bundles are tensed during a passive flexion of the knee joint, while PL-ACL and PM-PCL are kept relatively slacked. The reverse occurs during passive extension, where the posterior bundles of the ACL and PCL are tensed instead (Race and Amis, 1994; Harner et al., 1995; Siegel et al., 2012). Similar logic also applies to the collateral ligaments and particularly to the MCL ligament insertions over a wider range of areas on the femur and tibia (Robinson et al., 2005). A passive flexion tightens the anterior part of the ligament, while a passive extension tightens the posterior part. Different parts of all the ligaments, such as the superficial part of the MCL, further contribute to internal and external axial rotation of the tibia relative to the femur (Robinson et al., 2005), as well as a shear and moment loading of the knee. These various impact possibilities load the ligament sub-parts differently.

Several studies have tested the various functional parts of the ligaments (ACL, PCL, and MCL). Evaluations have focused on the biomechanical properties and the relative differences between the sub-ligaments to better understand their function (Woo et al., 1991; Race and Amis, 1994; Harner et al., 1995; Robinson et al., 2005), which has been necessary to truly distinguish them apart. This has usually been done by separating the functional parts and conducting a uniaxial tensile test of the sub-ligaments, with the purpose of better aligning the tensile load in parallel to the ligament fibers and avoiding partial ligament failure due to unevenly distributed load (discussed in Section 4.1). Previous studies found failure of knee ligaments to be influenced by the loading direction ( Woo et al., 1991; Mo et al., 2013). Various orientations and motions of the tibia relative to the femur will distribute the loads differently on the ligaments (Mo et al., 2013). With the purpose of estimating the risk of ligament injuries, optimizing ligament fiber recruitment to avoid sequential fiber failures in the ligaments could provide misleading or even erroneous IRFs, underestimating the true risk of injury at given knee positions by neglecting partial disruptions of the ligaments. To estimate the risk of injury, it could be of a larger value to evaluate each sub-ligament strain response for various relative positions of the bones and well-defined loading conditions.

Knee ligaments are rarely injured in isolation, but rather in combination with other adjacent soft tissues, such as the capsular ligaments and menisci, which also contribute to the passive restraint of the knee joint and therefore also influence the translational and rotational laxity of the knee joint after ligament injury (Willinger et al., 2021). The differences in knee kinematics between passive and active motions of the knee joint should be acknowledged while defining the loading conditions and the knee positions in injury scenarios. Muscle activation has been observed by Darcy et al. (2008) to influence the joint kinematics compared to passively induced motions, as well as compression forces such as those induced by the body weight during jump-landing and rapid sidestepping (Meyer and Haut, 2005; Bates et al., 2015). This suggests that muscle activation contributes to guiding the relative motion between the tibia and the femur (apart from the passive structures such as ligaments, menisci, and the geometrical construction of the bone plateau). While the active muscles affect the force response of the ligaments during injurious scenarios (Meyer and Haut, 2005), the same is not evident for the resulting failure strain outcome of the ligament *per se*. Both muscle activation and passive structures in the knee joint influence the relative motion between the tibia and the fibula, and the relative position between the bones at the time of injury might also deviate between passively and actively induced motion. This, in turn, results in different loading conditions of the knee ligaments, affecting the failure strain outcome. It is suggested to evaluate the relative positions of the bones during active motions in 3D motion capture recording, to capture the relative position between the tibia and the femur in all degrees of freedom, and to apply similar loading conditions according to the injurious scenario. This has been previously conducted by Bates et al. (2015) in the evaluation of the ACL and MCL responses to simulated landing scenarios using PMHS.

### 4.4 Specimen age correlation to failure strain

It is generally accepted that the biomechanical properties of tissues are affected by specimen age; however, only a few studies have been conducted that examine the correlation between age and biomechanical properties of human knee ligaments. Noyes and Grood. (1976) examined the tensile properties of the ACL between younger donors aged 16–26 years and older donors aged 48–86 years and found significant differences in the failure strain between the groups. However, the older specimens failed primarily by bone avulsion which, as the authors also state, does not represent the ligament behavior but rather that the bone appears to be the weakest link in the constellation. However, analysis of samples with pure ligament failure showed statistical significance of age-related decrease in elastic modulus, maximum stress, and strain energy. Woo et al. (1991) examined the effects of donor age on FATC between three age groups and found a significant age effect on the tensile strength, but with a reversed failure pattern compared to Noyes and Grood. (1976)—the younger specimens sustained bony avulsion, and the older specimens sustained mid-substance tear. Schmidt et al. (2019) studied the pediatric MCL, LCL, and PCL and found the mechanical properties to be considerably weaker compared to responses from the adult population, except for the ultimate failure strain responses, which were similar to the adult specimen literature. It should be noted, however, that these comparisons were partly made between the pediatric LIG structures and adult BLB structures, which essentially implies comparing two different specimen types with different responses to load. Moreover, ultrastructural differences of the cruciate ligaments have been observed by decreased collagen fiber diameter and increased concentration of the collagen fibril between mid-aged groups of 30–60 years and older people aged 60+ years. These are changes analyzed by the authors that could potentially make the ligaments more flexible for older people (Strocchi et al., 1996; Sargon et al., 2004). As the size of the collagen fibrils and the orientation of the collagen fibers have been shown to correlate to the ligaments’ mechanical response, it is within reason to believe that the age-related changes of the ligament microstructure could impact the risk of ligament failure. The dataset used in the current study provided IRFs with mixed donor ages, due to non-significance of the failure strains between the age groups below and above 40 years (Figure 2). The failure strains were considered the most crucial metric to develop risk functions for knee ligament injury prediction, due to the limited amount of failure data for all four ligaments in the state-of-the-art literature. Apart from providing only the average donor ages, many studies grouped a considerably wide span of ages, visualized by the large error bars in Figure 2. Although there are different conclusions in the literature on the relation of age to human ligament biomechanical responses, there is reason to suspect an age correlation with the ligaments’ biomechanical properties. For future age-dependent IRFs to be feasible, upcoming experimental studies need to provide the donor age for each specimen with a corresponding failure strain.

### 4.5 Recommendations for use and future improvements

The IRFs developed in this study are, to the best of the authors’ knowledge, the first attempt at knee ligament injury risk functions based on PMHS. The comparison between the dynamic and static IRFs for the ACL, PCL, and LCL has an expected behavior where the dynamic IRFs are translated to the left of the static IRFs (Figure 4). As this reflects the fact that the ligaments are weaker at higher dynamic rates, these IRFs are relevant in the analysis of ligament failure. The MCL IRFs, however, do not follow these expected tendencies, and the reliability of these IRFs is questionable.

Analyzing the injury risk of simulated knee ligament responses using HBMs, the boundary conditions will most often involve the ligament attached to the bones, making BLB IRFs the most relevant for traffic- and sport-related accident reconstructions. However, apart from providing IRFs, the current study highlights available data in the literature, and the IRFs of the LIG specimens are believed to be valuable while assessing the risk of failure for the isolated ligament. In addition, the two specimen types address a variety of failure modes and thus the generated IRFs of BLB and LIG should not be compared between the two specimen preparations. The dynamic IRFs are most suitable for use in the injury prediction of knee ligaments, as (low- and high-energy) accidents are not likely to cause injurious ligament loading in static rates. However, these IRFs are based on literature where most of the experiments used strain rates with magnitudes of 20%–100%/s, making them less suitable for potential injurious scenarios, which usually are caused by much higher impact rates on the ligaments. On the contrary, Bonner et al. (2015) found the material properties of porcine LCL to be rate-sensitive only up to 100%/s and statistically insignificant for strain rates beyond that.

Although both collateral ligaments have a similar restraining function of the knee joint, enough literature has not been found to support that the structural properties in the MCL and LCL are similar in the dynamic rate. Hence, a dynamic BLB IRF representing the MCL is needed for injury prediction of higher loading rates.

Assessing the injury risk against the developed IRFs herein should be conducted by using the crosshead strain of the whole ligament, as the IRF-based studies measured neither local strain rates nor local failure strains. Local failure strain datasets would facilitate more refined loading rate arrangements and thereby more advanced IRFs, as well as addressing covariates that most likely affect the material properties of ligaments, such as age and gender (Noyes and Grood, 1976; Woo et al., 1991; Chandrashekar et al., 2006; Winkelstein, 2013; Schmidt et al., 2019; Cho and Kwak, 2020). To conduct these analyses, future studies need to consistently specify the age and sex of each specimen.

The current study methodology was formulated to utilize data in the available literature to generate knee ligament IRFs, due to the insufficient amount of details correlated to the resulting failure strains provided. IRFs are ideally developed based on PMHS specimen-specific data from studies with comparable testing and measuring methods and a clear definition of ligament failure. Most experiments used to generate these IRFs deviated in all these factors, thereby adding complexity in finding uniform arrangements of the dataset. Consequently, a number of limitations exists and are worth considering.

- Due to data limitation, the current study has not accounted for the variety of study methods and test processes used between studies in the filtering process in the generation of IRFs due to the small sample size of the dataset for all the ligaments. The preservation method of the samples, the clamping methodology of the ligaments, preconditioning of the ligaments, the initial tensile angle of the ligament, and testing sample dimensions are examples of factors that could influence the resulting tensile failure strain—and should, ideally, be standardized.
- The IRFs were generated for failure at any location along the ligaments, and the failure regions (rupture at midsection, tibia insertion, or femur insertion) have not been distinguished.

The issue of incomplete data information is probably not unique for knee ligaments but is most likely also present for other tissue and body parts as well. The method of generating virtual failure strains from a mean and SD could, therefore, have potential as a valuable alternative method and should be prioritized to be validated. Furthermore, the provided IRFs need to be evaluated for their ability to predict injury, for example, by reconstructing known injurious and non-injurious scenarios using HBMs.

## 5 Conclusion

This study provides a first attempt at injury risk functions based on PMHS data for the four primary knee ligaments. Eight IRFs were developed for the ACL, MCL, and LCL—both in static and in more dynamic loading rates. Two IRFs were generated for the PCL in dynamic loading rates, while data for the PCL in static rates were insufficient. Specimen-specific failure strains were infrequently presented in the PMHS literature. For future improvements of the knee ligament IRFs, several important factors are required from the literature and upcoming experiments: comparable testing and strain-measuring methods, a clear definition of failure, and a transparent reporting of both specimen-specific results (e.g., strains) and specimen-specific characteristics (e.g., to account for age and sex differences).

## Data Availability

The original contributions presented in the study are included in the article/Supplementary Material; further inquiries can be directed to the corresponding author.
