# Supplementary material for: Injury risk functions for the four primary knee ligaments
Source: Front Bioeng Biotechnol. 2023 Oct 4;11:1228922. doi: 10.3389/fbioe.2023.1228922 (PMC10582698; doi:10.3389/fbioe.2023.1228922)
Supplement: Supplementary file 1 [file Table1.pdf]

APPENDIX A

TABLE A1. Mean failure strains ± SD as reported by studies conducting uniaxial tensile tests on either Bone-Ligament-Bone (BLB) specimens or dissected Ligaments (LIG). The studies have been grouped according to dynamic (red) or static (blue) tensile rate. “N” states the number of specimens used in the averaging and “Failure mode” specifies the injury mechanisms of the BLB specimens; (1) Mid-substance ligament failure; (2) Failure at ligament attachment site, or; (3) Bony avulsion/fracture. Empty boxes implies that the information was not presented. Studies meeting the inclusion criteria for the generation of risk functions are marked with a “X”.
*Information found in secondary sources (Race and Amis, 1994; Kerrigan et al., 2003; Balasubramanian, 2006).

Study	PMHS Sex [Male/Female]	PMHS Mean age [Range]	Mean strain [%]	SD [%]	N	Tensile rate	Specimen	Failure mode	Included
**ANTERIOR CRUCIATE LIGAMENT**									
Arnoux et al. (2002)	1M/3F	79 ± 14	*18-24*	-	7	1950 mm/s	BLB		
Butler et al. (1992) – AMB	2M/5F	26 ± 4	19.1	2.8	5	100 %/s	BLB	Ligament failure & insertion site	X
Butler et al. (1992) - ALB	2M/5F	26 ± 4	16.1	3.9	6	100 %/s	BLB	Ligament failure & insertion site	X
Butler et al. (1992) – PC	2M/5F	26 ± 4	15.2	5.2	6	100 %/s	BLB	Ligament failure & insertion site	X
Chandrashekar et al. (2006) – Male	8M	38 *(17 - 50)*	30.0	6.0	8	100 %/s	BLB	Ligament failure	X
Chandrashekar et al. (2006) – Female	9F	39 *(26 - 50)*	27.0	8.0	9	100 %/s	BLB	Ligament failure	X
Marieswaran et al. (2018) – Fresh samples	4M	*(60 - 70)*	56	4	7	80 %/s	BLB	All modes	

Study	PMHS Sex [Male/Female]	PMHS Mean age [Range]	Mean strain [%]	SD [%]	N	Tensile rate	Specimen	Failure mode	Included
**ANTERIOR CRUCIATE LIGAMENT *(cont.)***									
Marieswaran et al. (2018) – 10% formalin	4M	*(60 - 70)*	51	3	7	80 %/s	BLB	All modes	
Marieswaran et al. (2018) – Deep frozen	4M	*(60 - 70)*	70	5	7	80 %/s	BLB	All modes	
Marieswaran et al. (2021)	6M	*(60 – 70)*	45	3	6	300 %/s	BLB	Insertion site	X
Marieswaran et al. (2021)	6M	*(60 – 70)*	42	2	6	30 %/s	BLB	Ligament failure & insertion site	X
Noyes & Grood (1976)– Older	6M+	*(48 - 86)*	30	10	20	100%/s	BLB	Avulsion	
Noyes & Grood (1976) – Younger	3M/2F	*(16 - 38)*	44.3	8.5	6	100 %/s	BLB	Ligament failure	X
Van Dommelen et al. (2005) – aACL	7M	53 ± 10	18.0	2.8	4	54 ± 9.2 %/s	BLB	Ligament failure	X
Van Dommelen et al. (2005) – pACL	7M	53 ± 10	22.0	3.0	3	63 ± 3.4 %/s	BLB	Ligament failure	X
Kennedy et al. (1976)		62 *(20 - 75)*	30.8	2.3	10	2.083 mm/s	LIG		X
Kennedy et al. (1976)		62 *(20 - 75)*	35.8	2.8	10	8.33 mm/s	LIG		X
Jones et al. (1995) – Middle age		*(40 – 60)*			7	8.33 mm/s	BLB	Ligament failure	
Jones et al. (1995) – Older		*(61 - 82)*			21	8.33 mm/s	BLB	Ligament failure & insertion site	
Marieswaran et al. (2021)	6M	*(60 – 70)*	44	3	6	3 %/s	BLB	Insertion site	X
Paschos et al. (2010)	5M/5F	74 ± 16 *(44 - 88)*	42.7	18.5	10	1.5 mm/s	BLB	Ligament failure & insertion site	X
Trent et al. (1976)		*(29 - 55)*			6	0.833 mm/s	BLB	All modes	
Woo et al. (1991) – Younger	7M/2F	29 ± 5 *(22 – 35)*			9	3.33 mm/s	BLB	All modes	
Woo et al. (1991) – Middle age	3M/6F	45 ± 4 *(40 - 50)*			9	3.33 mm/s	BLB	All modes	
Woo et al. (1991) – Older	2M/7F	75 ± 10 *(60 - 97)*			9	3.33 mm/s	BLB	All modes	

Study	PMHS Sex [Male/Female]	PMHS Mean age [Range]	Mean strain [%]	SD [%]	N	Tensile rate	Specimen	Failure mode	Included
**POSTERIOR CRUCIATE LIGAMENT**									
Arnoux et al. (2002)	1M/3F	79 ± 14	*18-24*	-	5	1950 mm/s	BLB		
Butler et al. (1986) – Donor1	1F	30	14.6	4.6	3	100 %/s	BLB	Ligament failure	X
Butler et al. (1986) – Donor2	1M	30	14.0	2.4	2	100 %/s	BLB	Ligament failure	X
Butler et al. (1986) – Donor3	1F	21	18.9	2.9	3	100 %/s	BLB	Ligament failure	X
Prietto et al. (1988) *		23 ± 3 *(19 - 25)*	28.5	9.1	4	100 %/s	BLB		
Race & Amis (1994) – aPCL		75 ± 14	18.0	5.3	7	50 %/s	BLB		X
Race & Amis (1994)– pPCL		75 ± 14	19.5	5.4	10	50 %/s	BLB		X
Van Dommelen et al. (2005) – aPCL	7M	53 ± 10	18.0	2.3	2	45 ± 5.7 %/s	BLB	Ligament failure	X
Van Dommelen et al. (2005) – pPCL	7M	53 ± 10	14.0	1.4	3	49 ± 5.3 %/s	BLB	Ligament failure	X
Kennedy et al. (1976)		62 *(20 - 75)*	28.3	1.9	10	2.083 mm/s	LIG		X
Kennedy et al. (1976)		62 *(20 - 75)*	24.2	2.1	10	8.33 mm/s	LIG		X
Harner et al. (1995) – aPCL		52 *(30 - 83)*			14	3.33 mm/s	BLB	Ligament & insertion site	
Harner et al. (1995) – pPCL		52 *(30 - 83)*			14	3.33 mm/s	BLB	Ligament & insertion site	
Marinozzi et al. (1983) *		*(55 - 90)*	20.0	5.0	5	1.67 mm/s	BLB		
Trent et al. (1976)		*(29 - 55)*			6	0.83 mm/s	BLB	All modes	
Schmidt et al. (2019)	3M/2F	9	20.0	5.0	5	0.03 %/s	LIG		
**MEDIAL COLLATERAL LIGAMENT**									
Arnoux et al. (2002)	1M/3F	79 ± 14	*25 - 38*		5	1950 mm/s	BLB		
Kerrigan et al. (2003)	6M	56 ± 11	11.5	5.3	3	1205 ± 306 %/s	BLB	Ligament failure	X
Van Dommelen et al. (2005)	7M	53 ± 10	39.0	8.6	9	45 ± 5.7 %/s	BLB	Avulsion & Ligament failure	
Wilson et al. (2012)	4 M/5F	81 ± 11 *(62 – 92)*	14.81	4.97	9	20%/s	BLB	Avulsion & Insertion site	
Kennedy et al. (1976)		62 *(20-75)*	24.3	1.3	10	8.33 mm/s	LIG		X

Study	PMHS Sex [Male/Female]	PMHS Mean age [Range]	Mean strain [%]	SD [%]	N	Tensile rate	Specimen	Failure mode	Included
**MEDIAL COLLATERAL LIGAMENT *(cont.)***									
Kennedy et al. (1976)		62 *(20 - 75)*	23.0	2.4	10	2.083 mm/s	LIG		X
Cho & Kwak (2020)	7M/7F	77 ± 10 *(62 – 93)*			21	0.17 mm/s	BLB	All modes	
Kerrigan et al. (2003)	6M	56 ± 11	20.3	3.9	3	1.78 ± 0.35 %/s	BLB	Ligament failure	X
Marinozzi et al. (1983) *		*(55 - 90)*	13.0	6.0	5	1.7 mm/s	BLB		
Quapp and Weiss (1998)	9M/1F	62 ± 18	17.1	1.5	9	1%/s	LIG		X
Robinson et al. (2005) – sMCL		77 ± 5 *(72 - 89)*			8	16 mm/s	BLB	Ligament failure & insertion site	
Robinson et al. (2005) – dMCL		77 ± 5 *(72 - 89)*			8	16 mm/s	BLB	Ligament failure & insertion site	
Schmidt et al. (2019)	3M/2F	9	18.2	6.8	5	0.03 %/s	LIG		
Smeets et al. (2017)	10M/2F	74 ± 7	22.9	2.5	12	2 %/s	LIG		X
Trent et al. (1976)		*(29 - 55)*			4	0.83 mm/s	BLB	All modes	
Van Dommelen et al. (2005)	7M	53.4 ± 9.9	40.0	8.9	4	<0.001 %/s	BLB	Ligament failure	
Van Dommelen et al. (2005)	7M	53.4 ± 9.9	45.0	5.1	5	0.04±0.005 %/s	BLB	Ligament failure	
Wijdicks et al. (2010) –sMCL		54 *(27 - 68)*			16	0.33 mm/s	BLB	All modes	
Wijdicks et al. (2010) – dMCL		54 *(27 - 68)*			8	0.33 mm/s	BLB	All modes	
**LATERAL COLLATERAL LIGAMENT**									
Arnoux et al. (2002)	1M/3F	79 ± 14.4	*25- 38*		3	1950 mm/s	BLB		
Butler et al. (1986) – Donor1	1F	30	10.5	2.5	2	100 %/s	BLB	Ligament failure	X
Butler et al. (1986) – Donor2	1M	30	12.7	0.9	2	100 %/s	BLB	Ligament failure	X
Butler et al. (1986) – Donor3	1F	21	16.7	3.2	2	100 %/s	BLB	Ligament failure	X
Kerrigan et al. (2003)	6M	56 ± 11	10.5	4.6	3	1908 %/s	BLB	Ligament failure	X
LaPrade et al. (2005)	6M/2F	58 ± 11 *(40 - 77)*	16.0	5.0	8	100 %/s	BLB	Ligament failure	X

Study	PMHS Sex [Male/Female]	PMHS Mean age [Range]	Mean strain [%]	SD [%]	N	Tensile rate	Specimen	Failure mode	Included
**LATERAL COLLATERAL LIGAMENT *(cont.)***									
Van Dommelen et al. (2005)	7M	53.4 ± 9.9	18	1.6	8	37 ± 4.1 %/s	BLB	Avulsion & Ligament failure	
Wilson et al. (2012)	4 M/5F	81 ± 11 *(62 – 92)*	12.5	2.8	9	20 %/s	BLB	Ligament & insertion site	X
Cho & Kwak (2020)	7M/7F	77 ± 10 *(62 – 93)*			21	0.17 mm/s	BLB	All modes	
Kerrigan et al. (2003)	6M	56 ± 11	7.14	0.88	4	1.41 ± 0.07 %/s	BLB	Ligament failure	
Marinozzi et al. (1983) *		*(55-90)*	19.0	8.0	5	1.7 mm/s	BLB		
Smeets et al. (2017)	10M/2F	74 ± 7	41.0	9.9	11	2 %/s	LIG		X
Sugita & Amis (2001)		~70	16.1	2.5	9	3.33 mm/s	BLB		X
Trent et al. (1976)		*(29 - 55)*			5	0.83 mm/s	BLB	All modes	
Van Dommelen et al. (2005)	7M	53.4 ± 9.9	15.0	2.9	4	≤0.001 %/s	BLB	Ligament failure	X
Van Dommelen et al. (2005)	7M	53.4 ± 9.9	20.0	5.5	6	0.04 ±0.009 %/s	BLB	Ligament failure	X

REFERENCE LIST A1

Arnoux, P.J. *et al.* (2002) 'Knee ligament failure under dynamic loadings', *International Journal of Crashworthiness*, 7(3), pp. 255–268. Available at: https://doi.org/10.1533/cras.2002.0218.

Balasubramanian, S. (2006) *Posterior cruciate ligament (PCL) injury and repair: a biomechanical evaluation of the human knee joint under dynamic posterior loading ; kinematics and contact pressure measurements in normal, PCL deficient and PCL reconstructed knees*. Available at: http://gateway.proquest.com/openurl?url_ver=Z39.88-2004&rft_val_fmt=info:ofi/fmt:kev:mtx:dissertation&res_dat=xri:pqm&rft_dat=xri:pqdiss:3225820 (Accessed: 22 May 2023).

Butler, D.L. *et al.* (1992) 'Location-dependent variations in the material properties of the anterior cruciate ligament', *Journal of Biomechanics*, 25(5), pp. 511–518. Available at: https://doi.org/10.1016/0021-9290(92)90091-E.

Butler, D.L., Kay, M.D. and Stouffer, D.C. (1986) 'Comparison of material properties in fascicle-bone units from human patellar tendon and knee ligaments', *Journal of Biomechanics*, 19(6), pp. 425–432. Available at: https://doi.org/10.1016/0021-9290(86)90019-9.

Chandrashekar, N. *et al.* (2006) 'Sex-based differences in the tensile properties of the human anterior cruciate ligament', *Journal of Biomechanics*, 39(16), pp. 2943–2950. Available at: https://doi.org/10.1016/j.jbiomech.2005.10.031.

Cho, H.-J. and Kwak, D.-S. (2020) 'Mechanical Properties and Characteristics of the Anterolateral and Collateral Ligaments of the Knee', *Applied Sciences*, 10(18), p. 6266. Available at: https://doi.org/10.3390/app10186266.

Harner, C.D. *et al.* (1995) 'The human posterior cruciate ligament complex: an interdisciplinary study. Ligament morphology and biomechanical evaluation', *The American Journal of Sports Medicine*, 23(6), pp. 736–745. Available at: https://doi.org/10.1177/036354659502300617.

Jones, R.S. *et al.* (1995) 'Mechanical properties of the human anterior cruciate ligament', *Clinical Biomechanics (Bristol, Avon)*, 10(7), pp. 339–344. Available at: https://doi.org/10.1016/0268-0033(95)98193-x.

Kennedy, J.C. *et al.* (1976) 'Tension studies of human knee ligaments. Yield point, ultimate failure, and disruption of the cruciate and tibial collateral ligaments', *JBJS*, 58(3), p. 350.

Kerrigan, J.R. *et al.* (2003) 'Rate-sensitive Constitutive and Failure Properties of Human Collateral Knee Ligaments'.

LaPrade, R.F. *et al.* (2005) 'Mechanical properties of the posterolateral structures of the knee', *The American Journal of Sports Medicine*, 33(9), pp. 1386–1391. Available at: https://doi.org/10.1177/0363546504274143.

Marieswaran, M. *et al.* (2018) 'Effect of preservation methods on tensile properties of human femur-ACL-tibial complex (FATC) – a cadaveric study on male subjects', *Acta of Bioengineering and Biomechanics*, Vol. 20(nr 3). Available at: https://doi.org/10.5277/ABB-01134-2018-03.

Marieswaran, M. *et al.* (2021) 'A cadaveric study on the rate of strain-dependent behavior of human anterior cruciate ligament', *Acta of Bioengineering and Biomechanics*, 23(1). Available at: https://doi.org/10.37190/ABB-01672-2020-05.

Marinozzi, G., Pappalardo, S. and Steindler, R. (1983) 'Human knee ligaments: mechanical tests and ultrastructural observations', *Italian journal of orthopaedics and traumatology*, 9(2), pp. 231–240.

Noyes, F.R. and Grood, E.S. (1976) 'The strength of the anterior cruciate ligament in humans and Rhesus monkeys', *JBJS*, 58(8), p. 1074.

Paschos, N.K. *et al.* (2010) 'Cadaveric Study of Anterior Cruciate Ligament Failure Patterns Under Uniaxial Tension Along the Ligament', *Arthroscopy: The Journal of Arthroscopic & Related Surgery*, 26(7), pp. 957–967. Available at: https://doi.org/10.1016/j.arthro.2009.12.013.

Prietto, M.P. *et al.* (1988) 'Tensile strength of the human posterior cruciate ligament (PCL)', *Trans Orthop Res Soc*, 13(195), pp. 736–745.

Quapp, K.M. and Weiss, J.A. (1998) 'Material characterization of human medial collateral ligament', *Journal of Biomechanical Engineering*, 120(6), pp. 757–763. Available at: https://doi.org/10.1115/1.2834890.

Race, A. and Amis, A.A. (1994) 'The mechanical properties of the two bundles of the human posterior cruciate ligament', *Journal of Biomechanics*, 27(1), pp. 13–24. Available at: https://doi.org/10.1016/0021-9290(94)90028-0.

Robinson, J.R., Bull, A.M.J. and Amis, A.A. (2005) 'Structural properties of the medial collateral ligament complex of the human knee', *Journal of Biomechanics*, 38(5), pp. 1067–1074. Available at: https://doi.org/10.1016/j.jbiomech.2004.05.034.

Schmidt, E.C. *et al.* (2019) 'Mechanical and Microstructural Properties of Native Pediatric Posterior Cruciate and Collateral Ligaments', *Orthopaedic Journal of Sports Medicine*, 7(2), p. 2325967118824400. Available at: https://doi.org/10.1177/2325967118824400.

Smeets, K. *et al.* (2017) 'Mechanical Analysis of Extra-Articular Knee Ligaments. Part One: Native knee ligaments', *The Knee*, 24(5), pp. 949–956. Available at: https://doi.org/10.1016/j.knee.2017.07.013.

Sugita, T. and Amis, A.A. (2001) 'Anatomic and biomechanical study of the lateral collateral and popliteofibular ligaments', *The American Journal of Sports Medicine*, 29(4), pp. 466–472. Available at: https://doi.org/10.1177/03635465010290041501.

Trent, P.S., Walker, P.S. and Wolf, B. (1976) 'Ligament Length Patterns, Strength, and Rotational Axes of the Knee Joint.', *Clinical Orthopaedics and Related Research (1976-2007)*, 117, p. 263.

Van Dommelen, J.A.W. *et al.* (2005) 'Characterization of the Rate-Dependent Mechanical Properties and Failure of Human Knee Ligaments', in. *SAE 2005 World Congress & Exhibition*, pp. 2005-01–0293. Available at: https://doi.org/10.4271/2005-01-0293.

Wijdicks, C.A. *et al.* (2010) 'Structural properties of the primary medial knee ligaments', *The American Journal of Sports Medicine*, 38(8), pp. 1638–1646. Available at: https://doi.org/10.1177/0363546510363465.

Wilson, W.T. *et al.* (2012) 'Comparative analysis of the structural properties of the collateral ligaments of the human knee', *The Journal of Orthopaedic and Sports Physical Therapy*, 42(4), pp. 345–351. Available at: https://doi.org/10.2519/jospt.2012.3919.

Woo, S.L. *et al.* (1991) 'Tensile properties of the human femur-anterior cruciate ligament-tibia complex. The effects of specimen age and orientation', *The American Journal of Sports Medicine*, 19(3), pp. 217–225. Available at: https://doi.org/10.1177/036354659101900303.
