# Supplementary material for: Injury risk functions for the four primary knee ligaments
Source: Front Bioeng Biotechnol. 2023 Oct 4;11:1228922. doi: 10.3389/fbioe.2023.1228922 (PMC10582698; doi:10.3389/fbioe.2023.1228922)
Supplement: Supplementary file 2 [file Table2.pdf]

APPENDIX B

TABLE B1. Resulting p-value for the goodness of fit in the Anderson-Darling test and the root-mean-square-error (RMSE) for three of the best fitting distributions of the chosen generation group. The adequate generation group was chosen based on the smallest range of the statistical parameters in the cumulative distribution function. Yellow marks the chosen distribution based on p-value, RMSE and visual observations.

	Gamma distribution		Log-Logistic distribution		Log-Normal distribution		Weibull distribution		Generation
	p-Value	RMSE [%]	p-Value	RMSE [%]	p-Value	RMSE [%]	p-Value	RMSE [%]	
**ACL-dynamic-BLB**	0.836	4.37	0.851	4.04	0.948	3.53	0.623	5.00	100
**ACL-static-BLB**	0.942	7.38	0.907	7.74	0.900	7.76	0.905	7.93	-
**ACL-dynamic-LIG**	0.987	4.65	0.976	4.70	0.995	4.22	0.966	5.54	100
**PCL-dynamic-BLB**	1	1.49	0.999	2.33	1	1.51	0.999	3.39	500
**PCL-dynamic-LIG**	0.992	4.03	0.980	4.57	0.994	3.76	0.995	4.68	500
**MCL-dynamic-LIG**	0.997	2.92	0.999	3.47	0.999	3.16	1	3.01	50
**MCL-static-LIG**	0.850	4.68	0.908	4.83	0.916	4.57	0.603	6.27	100
**LCL-dynamic-BLB**	0.993	3.13	0.999	2.59	0.913	3.81	0.954	4.86	500
**LCL-static-BLB**	0.999	4.26	1	2.86	1	3.51	0.855	6.79	100
**LCL-static-LIG**	0.884	13.0	0.971	10.2	0.939	12.2	0.650	14.2	-
